# The Alkaline Resistance of Waterborne Acrylic Polymer/SiO_2_ Nanocomposite Coatings

**DOI:** 10.1155/2022/8266576

**Published:** 2022-04-22

**Authors:** Hoang Thi Huong Thuy, Hoang Thu Ha, Nguyen Thien Vuong, Tuan Anh Nguyen

**Affiliations:** ^1^Hong Duc University, 565 Quang Trung, Dong Ve, Thanh Hoa, Thanh Hoa, Vietnam; ^2^VNU-University of Education, Vietnam National University, Hanoi, Vietnam; ^3^Institute for Tropical Technology, VAST, 18 Hoang Quoc Viet, Cau Giay, Hanoi, Vietnam; ^4^Graduate University of Science and Technology, VAST, 18 Hoang Quoc Viet, Cau Giay, Hanoi, Vietnam

## Abstract

This study presents a study on the influence of nano-SiO_2_ on the alkaline resistance of waterborne acrylic coating using some analysis methods such as FT-IR and UV-Vis spectroscopy, combined with FE-SEM analysis and monitoring weight and adhesion changes during exposure to the saturated Ca(OH)_2_ alkaline environment. The obtained results indicated that the alkaline resistance of acrylic coating enhanced appreciably when adding 2.5 wt% of nano-SiO_2_. Under the impact of the saturated Ca(OH)_2_ solution for 20 days of immersion, nanocomposite coating containing 2.5 wt.% of nano-SiO_2_ was only decreased by 3.6% of the weight and 15.4% of the adhesion, while the neat acrylic coating (without nano-SiO_2_) seriously reduced 25.4% of the weight and 39.1% of the adhesion.

## 1. Introduction

Waterborne acrylic polymers, which can be synthesized from acrylic monomers (methyl acrylate, butyl acrylate, 2-ethylhexyl acrylate, acrylic acid, and methyl methacrylate), are commonly used to produce protective and decorative paints for buildings due to their good mechanical properties and high photochemical resistance [1]. However, during long-term exposure to the alkaline environment of concrete, the acrylic coatings can be degraded, namely, decreasing their surface morphology, adhesion, and protection ability [2].

Recently, due to the advances in nanoscientific and nanotechnological sectors, many smart functional coatings have been simply fabricated by adding nanoparticles into their formulations. The introduction of nanoparticles (nano-TiO_2_ [3], nano-ZnO [4], nano-Fe_3_O_4_ [5], and nano-SiO_2_, [5–12]) into the polymer substrates created various advanced functions such as UV shielding, self-cleaning, and antimicrobials of the coating. Among them, silica nanoparticles have been most widely applied [13, 14]. For example, UV and water resistance of the waterborne acrylic resin improved by introducing the modified nano-TiO_2_/SiO_2_ composite particles [15]. In addition, the coating containing SiO_2_ nanoparticles exhibited remarkable thermal and anticorrosion properties [16, 17]. As proved by some previously published works, the mechanical properties, weather resistance, and acid and alkaline resistance of acrylic polyurethane were reinforced by the addition of 2.5 wt% of SiO_2_ nanoparticles into the polymer matrix [6, 7].

In case of cement and concrete studies [18–24], nano-SiO_2_ could enhance the concrete workability and strength [19] to improve the resistance to water penetration [20] and to prevent the leaching of calcium [21] out of concrete. Nano-SiO_2_ could not only act as a nanofiller to enhance the microstructure but also act as an activator to promote pozzolanic reactions [23]. Thus, we expect that the incorporation of nano-SiO_2_ into the acrylic polymer matrix could prevent the leaching of calcium/Ca(OH)_2_ out of coating. Therefore, when Ca(OH)_2_ penetrates into the coating, it might react with nano-SiO_2_ to form the C-S-H metastable phase (calcium silicate hydrate, CaO-SiO_2_-H_2_O) that further reinforce the acrylic coating (more dense).

In this study, we will report our research results on the effect of silica nanoparticles on the alkaline resistance of waterborne acrylic coating using some analysis methods such as FT-IR and UV-Vis spectroscopy, FE-SEM, and weight and adhesion changes monitoring during the exposure to the Ca(OH)_2_ alkaline environment.

## 2. Experimental

### 2.1. Materials

Acrylic polymer dispersion Plextol R4152, which has a total solids content of 50%, pH of 7–8.5, a viscosity of 500–3000 cps, and particle size of 0.13 *µ*m, was purchased from Synthomer Company. Coalescing agent is Texanol (2,2,4-trimethyl-1,3-pentanediol monoisobutyrate), purchased by Dow Chemical Company (United States). Silica nanoparticles with a particle size of 10–20 nm were synthesized from rice husk ash [6]. All of the purchased reagents were used without further puriﬁcation.

### 2.2. Sample Preparation

#### 2.2.1. Preparation of Paint Formulations

Silica nanoparticles were selected as 2.5 wt% (by weight on total solid resin) according to the optimal content for our previous acrylic polyurethane/SiO_2_ nanocomposite coating [6].

The first mixture consisting of silica nanoparticles (2.5 g) dispersed into deionized water (40 g) was carried out under ultrasonic vibration using a supersonic equipment TPC-25 (Switzerland) for 60 min. By the same way, the second mixture containing Texanol (3 g) and acrylic resin R4152 (200 g) was prepared. Thereafter, the first mixture was mixed with the second mixture under ultrasonic dispersion for 3 hours at room temperature.

#### 2.2.2. Preparation of Paint Films

The paint films for IR, UV-Vis, FE-SEM, and weight loss analyses were prepared on glass panels with a size of (100 mm × 70 mm × 2 mm) using the Quadruple Film Applicator Erichsen Model 360 with a wet film thickness of 60 *µ*m which is equivalent, corresponding to the dried coating thickness of 25 ± 2 *µ*m. The samples for IR measurement were peeled away from the glass panels and then attached on stainless steel windows. The samples for measuring the adhesion were made as reported in previous article [25]. Finally, the coatings were dried at room temperature for 7 days before analysis and testing.

### 2.3. Devices and Methods

#### 2.3.1. Transmission Electron Microscopy (TEM) and Field Emission Scanning Electron Microscope (FE-SEM)

The morphology of nano-SiO_2_ and nanocomposite was analyzed using S-4800 FE-SEM (Hitachi, Japan) and TEM-JEM 2100 (Jeol, Japan).

#### 2.3.2. FT-IR and UV-Vis Characteristics

The FT-IR spectra of the coatings before and after immersion in alkaline solution were recorded by the Thermo Nicolet Nexus 670 FT-IR Spectrometer (United States).

The change of chromophore was measured and monitored in a GBC Cintra 40 UV-Visible Spectrometer (United States).

#### 2.3.3. Determination of Weight Loss

Coatings before and after 20 days immersion were clearly washed and dried at 60°C until their weight remained constant. The remaining weight of paint film was determined as follows:

(1) $$\begin{array}{c} \text{Remaining weight}\left(\%\right)=\frac{m_{t}}{m_{0}}\times 100, \end{array}$$

where *m*_0_ represents the weight of initial samples and *m*_*t*_: represents the weight of samples after 20 days of immersion in alkaline solution.

#### 2.3.4. Adhesion Measurement

The adhesion of the paint samples was measured according to ASTM D 4541 in the same tool as presented in previously published work [25].

#### 2.3.5. Durability of Coatings in Alkaline Environment

The water-based coatings are used in construction, exposed to weakly alkaline environments of concrete; thus, its alkaline resistance was tested according to the TCVN 8653-3: 2012 (Vietnam Standard 8653-3: 2012) by immersing the samples in the saturated Ca(OH)_2_ solution for 20 days. The coating samples were washed with deionized water and dried at 60°C in vacuum oven in 24 hours before further measurement.

## 3. Results and Discussion

### 3.1. Morphology of Silica Nanoparticles

**Figure 1:**
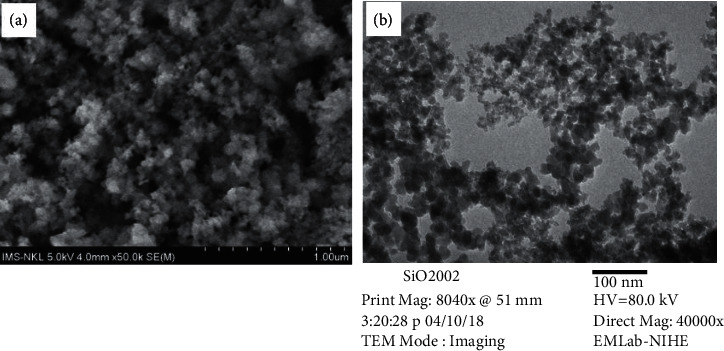
FE-SEM (a) and TEM (b) images of SiO_2_ nanoparticles.

FE-SEM and TEM images of the initial SiO_2_ nanoparticles have been taken (Figure 1) to observe their size and agglomerations.

From Figure 1, with the magnification of about 40.000–50.000 times, it is seen that the size of SiO_2_ nanoparticles was fairly uniform with the diameter of around 15 ± 5 nm. These nanoparticles were not scattered but aggregated together into highly porous structures.

### 3.2. FT-IR Spectra Study

**Figure 2:**
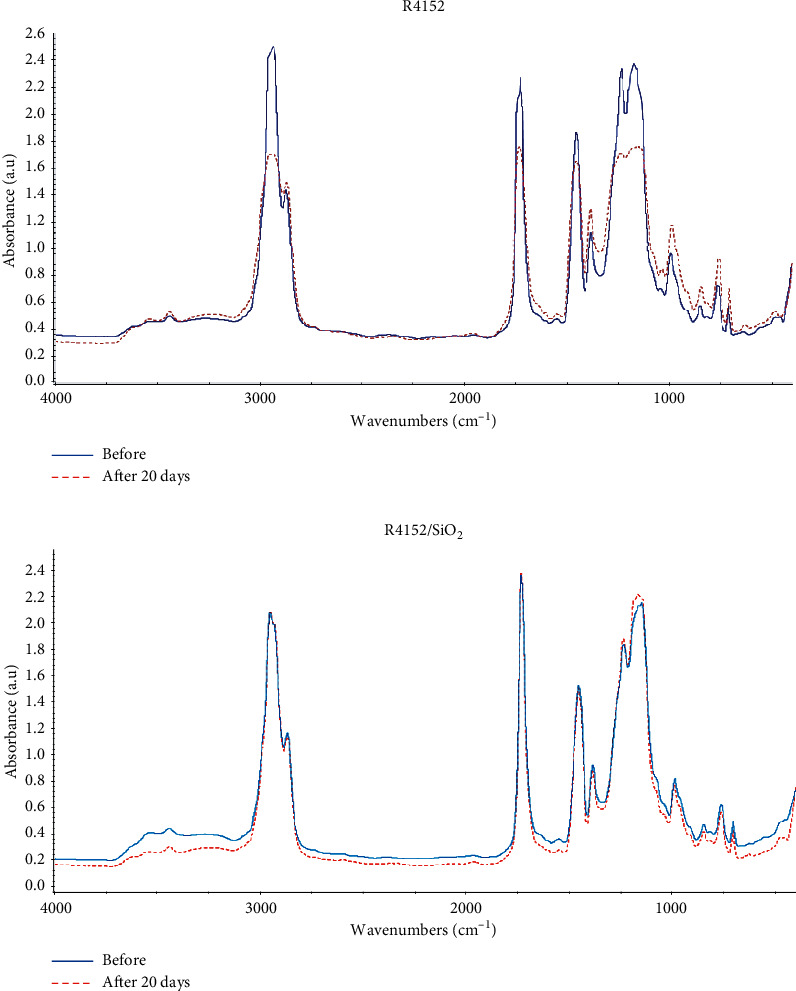
IR spectra of acrylic coating without nano-SiO_2_ (R4152) and with 2.5 wt% nano-SiO_2_ (R4152/SiO_2_) before and after 20 days of exposure in the saturated Ca(OH)_2_ environment.

IR spectroscopy is commonly applied to detect early the changes of polymer materials during the ageing process [24].  Figure 2 and Table 1 show the IR absorption bands of the acrylic coating without nano-SiO_2_ (R4152) and the acrylic coating containing 2.5 wt% nano-SiO_2_ (R4152/SiO_2_) before and after immersion in the saturated Ca(OH)_2_ solution for 20 days.

Comparing the IR spectra of acrylic coating with and without nano-SiO_2_ indicated that there are still a full range of absorption bands characterized for acrylic polymer. For example, the band at about 2960–2850 cm^−1^ was related to the presence of the stretching vibrations of C–H bonds in acrylic polymer. Additionally, the bands at 1730 cm^−1^ were attributed to the stretching vibrations of C=O in ester groups.

However, there was a clear difference between the IR spectra of the coatings before and after immersion. In the coating without nano-SiO_2_ (R4152), it can be realized that after 20 days of immersion, the intensity vibrations of the alkane C–H groups, ester C=O, and C–O groups decreased strongly while not appearing the new functional groups. This suggests that the low-molecular-weight compounds (including polymers) had been released out of coating during the immersion process. By contrast, in the acrylic coating containing nano-SiO_2_ (R4152/SiO_2_), the intensity vibrations characterized for all functional groups were insignificantly changed.

All the results in IR spectra showed that SiO_2_ nanoparticles were successfully loaded into the acrylic by the above-prepared method. Besides, the nanocomposite coating samples (R4152/SiO_2_) had less alteration and less degradation due to the better shielding ability to the alkaline medium.

**Figure 3:**
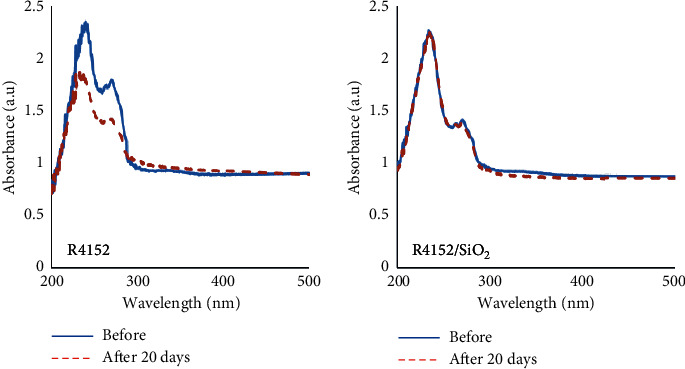
UV-Vis spectra of acrylic coating without SiO_2_ and the acrylic coating with 2.5 wt%.

After immersion in the saturated Ca(OH)_2_ solution for 20 days (Figure 3), the intensity of both bands in the R4152 coating was decreased sharply. Meanwhile, the intensity of both absorbance bands in R4152/SiO_2_ coating can be considered unchanged. It could be concluded that the nano-SiO_2_ had filled into defects and holes in the composite coating, against the penetration of the alkaline environment. The alkaline resistance of the nanocomposite coating in this work is higher than that of the coating based on the GP 1106 styrene acrylic emulsion [29].

### 3.3. UV-Vis Spectra of the Coating

The UV-Vis spectra of the coating without and with nano-SiO_2_ before and after 20 days of immersion in the saturated Ca(OH)_2_ solution are shown in Figure 3.

This figure shows that when adding nano-SiO_2_, two absorption peaks (at the wavelength of 237 nm and 273 nm) characterized for the R4152 acrylic polymer was almost unchanged after 20 days of immersion, while those of the coating without nano-SiO_2_ were significantly decreased.

### 3.4. Weight Loss of the Coating

**Figure 4:**
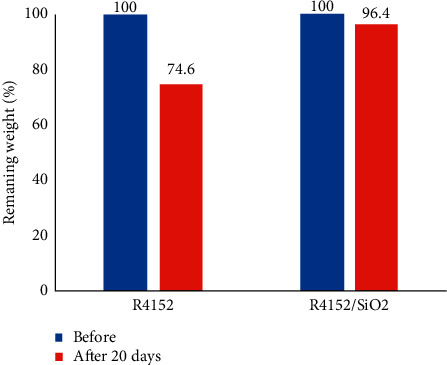
The remaining weight of acrylic coatings without nano-SiO_2_ (R4152) and with 2.5 wt% nano-SiO_2_ (R4152/SiO_2_) before and after immersion in the saturated Ca(OH)_2_ environment for 20 days.

Besides estimating the chemical structural alteration by IR spectroscopy, the degradation of coating was also evaluated by quantifying the weight changes of the coating in the process of SiO_2_ before and after 20 days of exposure to the saturated Ca(OH)_2_ environment exposure to the saturated Ca(OH)_2_ environment. The weight loss of the acrylic coating with and without nano-SiO_2_ during the alkali exposure is shown in Figure 4.

It can be seen that after 20 days of the immersion, the nanocomposite coating (R4152/SiO_2_) lost only 3.6%, while the acrylic coating without nano-SiO_2_ (R4152) decreased 25.4% of weight. The result indicated that the nanocomposite coating had the better alkaline resistance.

### 3.5. Morphological Study

**Figure 5:**
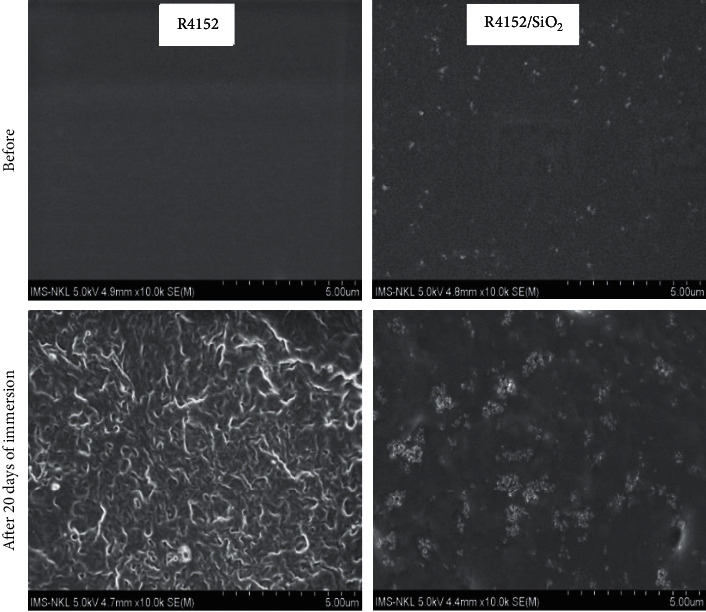
FE-SEM images of the acrylic coating without nano-SiO_2_ (R4152) and with 2.5 wt% nano-SiO_2_ (R4152/SiO_2_) before and after immersion in the saturated Ca(OH)_2_ environment for 20 days.

The morphology of the coating samples before and after the immersion was observed by FE-SEM images (Figure 5).

It is clear that after 20 days, under the impact of alkaline solution, the surface of the acrylic coating without nano-SiO_2_ (R4152) had a severe deformation, while the nanocomposite coating (R4152/SiO_2_) almost remained in its original form. There are only a few microholes originating from some SiO_2_ nanoparticles that peeled off the coating under the influence of the saturated Ca(OH)_2_ solution.

The obtained results in R4152/SiO_2_ coating could be interpreted as follows: the holes in the coating were filled by SiO_2_ nanoparticles; therefore, it is difficult for alkaline solution to penetrate into the acrylic coating containing nano-SiO_2_, so the coating retains its morphology. In contrast, for the coating without nano-SiO_2_ (R4152), the alkaline solution is easy to penetrate deeply into the coating, leading to swelling and deformation.

### 3.6. Reduction in Adhesion of the Coating

**Figure 6:**
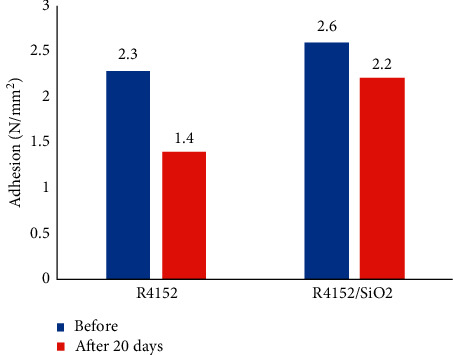
Adhesion of the acrylic coating without nano-SiO_2_ and the acrylic coating with 2.5 wt% of nano-SiO_2_ before and after immersion in the saturated Ca(OH)_2_ environment for 20 days.

The adhesion of coatings on the concrete substrate is one of the factors to evaluate the degradation of the coating during alkaline hydrolysis. The adhesion of the acrylic coating without nano-SiO_2_ (R4152) and the nanocomposite coating (R4152/SiO_2_) before and after immersion in the saturated Ca(OH)_2_ solution for 20 days are shown in Figure 6.

It is seen from this figure, under the influence of alkaline solution for 20 days, the adhesion of R4152/SiO_2_ coating decreased by 15.4% (from 2.6 to 2.2 N/mm^2^) while that of the R4152 coating (without nano-SiO_2_) lost by 39.1%. It indicated that the presence of SiO_2_ nanoparticles had significantly improved the adhesion of the coating under the effect of alkaline environment.

## 4. Conclusions

The research results of the chemical structures, the weight loss, the morphology, and the adhesion of the acrylic coating without and with 2.5 wt% of SiO_2_ nanoparticles after immersion in the saturated Ca(OH)_2_ solution indicated clearly that the alkaline resistance of acrylic coating was enhanced appreciably when adding 2.5 wt% of nano-SiO_2_. Under the influence of the saturated Ca(OH)_2_ solution for 20 days, nanocomposite coating containing 2.5 wt% of nanosilica was changed slightly, such as only lost 3.6% of the weight and 15.4% of the adhesion, while the neat acrylic coating was seriously degraded by 25.4% of weight and 39.1% of adhesion.

This study is the first work which explains how SiO_2_ nanoparticles can enhance the alkaline resistance of waterborne acrylic polymer. SiO_2_ nanoparticles can play the dual roles as nanofillers to enhance the mechanical properties of acrylic polymer coating and as self-healing nanoadditives to enhance the alkaline resistance of waterborne acrylic coating, when applied in the surface of concrete structures.

## Figures and Tables

**Table 1:** The absorption bands in the IR spectra of acrylic coating containing 2.5 wt% nano-SiO_2_ before and after immersion in the saturated Ca(OH)_2_ environment for 20 days.

Absorption (cm^−1^)	Functional group	0 day	20 days	Conclusion	
				Intensity	Changes
3550–3443	O–H stretching (alcohol, acid)	+	+	Weak	—
2960–2850	C–H stretching (alkane)	+	+	Strong	Decrease
1730	C=O stretching (ester)	+	+	Strong	Decrease
1470	C–H bending (alkane)	+	+	Strong	Decrease
1387	C–H bending (alkane)	+	+	Medium	Decrease
1250–1165	C–O stretching (ester)	+	+	Strong	Decrease

“+”, absorption.

## Data Availability

The data used to support this study are included within the article.
